# A Rare Case of Guillain-Barré Syndrome Associated With SARS-CoV-2 Infection Requiring Mechanical Ventilation

**DOI:** 10.7759/cureus.25810

**Published:** 2022-06-10

**Authors:** Rahul Prakash Rane, Ashish Jain, Khandakar M Hussain, Sarvesh Naik, Asna Shahab

**Affiliations:** 1 Internal Medicine, Conemaugh Memorial Medical Center, Johnstown, USA

**Keywords:** post covid gbs/ atm, guillain-barre syndrome (gbs), neuromuscular diseases, invasive mechanical ventilation, sars-cov-2, covid-19

## Abstract

Coronavirus disease 2019 (COVID-19) has become a worldwide pandemic since the first case of severe acute respiratory syndrome coronavirus 2 (SARS-CoV-2) infection was identified in December 2019. Numerous neurological consequences have been reported with COVID-19 infection and its approved vaccines. However, Guillain-Barré syndrome (GBS) is a rare neurological complication associated with COVID-19 infection. This case report describes a 62-year-old female with a three-week history of COVID-19 infection who presented with symmetric polyneuropathy in bilateral lower extremities that progressed to involve bilateral upper extremities and skeletal muscles of respiration, resulting in respiratory distress and necessitating intubation and mechanical ventilation. Cerebrospinal fluid (CSF) analysis revealed albumino-cytologic dissociation, and our patient met the National Institute of Neurological Disorders and Strokes (NINDS) criteria for diagnosing Guillain-Barré Syndrome, making GBS to be the most likely diagnosis. This case report aims to strengthen the association of GBS with COVID-19 infection and describes the hospital course of GBS.

## Introduction

Guillain-Barré Syndrome (GBS) is a common cause of acute, acquired polyneuropathy. GBS is often precipitated by a preceding infection. While *Campylobacter jejuni* (*C. jejuni*) continues to be the most prevalent trigger preceding GBS [1], emerging evidence has linked antecedent COVID-19 infection to GBS [2,3]. Classically, GBS presents as progressive and symmetric lower limb muscle weakness. The muscle weakness eventually progresses over a period of two to four weeks to involve the upper limbs in a majority of the patients [4]. However, the involvement of respiratory muscles prompting ventilatory support develops in less than 30 percent of the patients, therefore It is essential to monitor forced vital capacity (FVC) and negative inspiratory force (NIF) in patients to detect the possibility of hypercapnia with hypoxia and need for mechanical ventilation [5]. Absent or decreased deep tendon reflexes in either upper or lower limbs is a cardinal finding in GBS [4]. In summary, although GBS is a rare complication of COVID-19 infection, it is vital to maintain vigilance so that any such complication may be accurately identified. Our case report aims further to strengthen the association of GBS with COVID-19 infection and provides a detailed hospital course and management of GBS. 

## Case presentation

A 62-year-old woman with a three-week history of COVID-19 infection treated with the center of disease control (CDC) guided outpatient treatment presented with progressive weakness and numbness in bilateral lower extremities at an outside facility. The physical examination revealed reduced motor strength in bilateral lower extremities. Initial laboratory examinations were performed to identify the common causes of polyneuropathies. The values of vitamin B12, folate, thyroid panel, and hemoglobin A1c were all within normal parameters. In addition, the patient's SARS-CoV-2 PCR (polymerase chain reaction), HIV, and Lyme disease antibody tests were negative (Table 1).

**Table 1:** Laboratory workup for common causes of polyneuropathy such as diabetes mellitus, HIV infection, vitamin B12 deficiency, etc. was negative TSH- Thyroid Stimulating Hormone; CRP- C Reactive Protein; AB- Antibody; ANA- Antinuclear antibody; AG- Albumin: Globulin; MuSK- Muscle-specific tyrosine kinase; AChR- Acetylcholine receptor; NMO- Neuromyelitis Optica; IgG/IgM- Immunoglobulin G/ Immunoglobulin M; PCR- Polymerase chain reaction; HIV- Human immunodeficiency Virus; Hep- Hepatitis; HSV- Herpes Simplex Virus; HTLV- Human T-lymphotropic virus

Component.	Result	Ref Range & Units
TSH	1.05	0.35 - 4.94 uIU/mL
Free T4	1.69	0.89 - 1.76 ng/dL
Vitamin B-12	631	211 - 911 pg/mL
Folate	8.54	>5.38 ng/mL
Vit D, 25-Hydroxy	22.7	0.0 - 150.0 ng/mL
CRP	14.5	0.0 - 5.0 mg/L
Sed Rate	14	0 - 30 mm/hr
Procalcitonin	0.05	0.00 - 0.05 ng/mL
Ferritin	217.7	2.0 - 290.0 ng/mL
D-Dimer	0.37	<0.50 mg/L FEU
Serum protein electrophoresis		
Albumin Electrophoresis	4.26	3.38 - 4.26 g/dL
Alpha 1	0.38	0.16 - 0.34 gm/dL
Alpha 2	0.94	0.58 - 0.94 gm/dL
Beta	1.15	0.94 - 1.42 gm/dL
Gamma Globulin	1.78	0.46 - 1.86 gm/dL
AG Ratio	1	0.80 - 1.50
Rheumatoid Factor	Negative	Negative
ANA Screen	Negative	Negative
MuSK Antibodies	<1.0	U/mL
Myasthenia Gravis Profile		
AChR Binding Abs, Serum	0.07	0.00 - 0.24 nmol/L
AChR Blocking Abs, Serum	15	0 - 25 %
AChR Modulating Abs, Serum		Test Could not be performed.
NMO IgG Autoantibodies	<1.5	0.0 - 3.0 U/mL
Lyme Disease PCR	Negative	Negative
Lyme Total IgM/IgG Ab	Non-Reactive	Non-Reactive
Lyme Total IgM/IgG Ab Index	0.2	<=0.8 AI
HIV ANTIGEN/ANTIBODY	Negative	Negative
Hep A IgM	Negative	Negative, Borderline
Hep B Core IgM	Negative	Negative, Borderline
Hepatitis B Surface Ag	Negative	Negative
Hepatitis C Ab	Negative	Negative, Borderline
HSV, IgM I/II Combination	<0.91	0.00 - 0.90 Ratio
HSV 1 IgG, Type Spec	8.05	0.00 - 0.90 index
HSV 2 IgG, Type Spec	<0.91	0.00 - 0.90 index
HTLV-I/II Antibodies, Qual	Negative	Negative
Syphilis IgG AB	Non-Reactive	Non-Reactive, Equivocal

**Figure 1:**
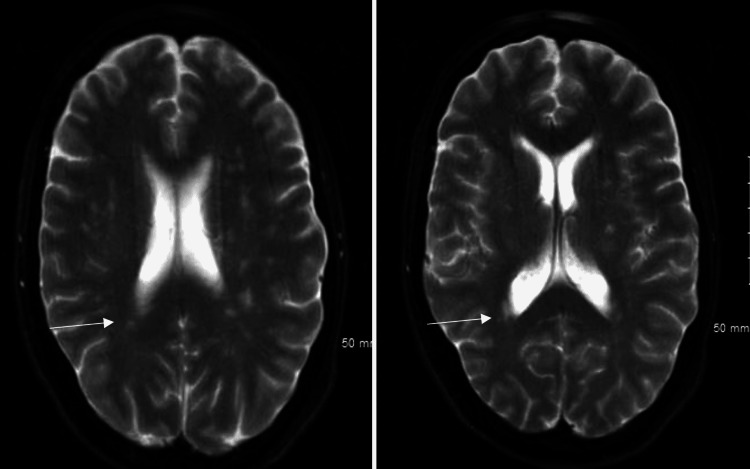
MRI of the head in transverse plane showing periventricular hyper-intensities.

The patient was empirically started on steroids which did not alleviate her symptoms at the outside facility, and thus, the patient was transferred to our hospital for further neurological evaluation. On arrival at our medical facility, it was noted that the patient's weakness progressively worsened to involve upper extremities, she started developing difficulty with her speech, and physical examination showed areflexia at the knees and ankles bilaterally. The patient's neurological symptoms deteriorated rapidly within a few hours, and she developed acute hypoxic respiratory failure. This necessitated intubation, and mechanical ventilation after an attempt of noninvasive positive pressure ventilation failed (NIPPV). The MRI of the head revealed periventricular hyperintensities suggestive of multiple sclerosis (Figure 1). However, Multiple sclerosis was low on differential diagnosis, given that the patient did not respond to steroids given at the outside facility and the rapid advancement of neurological deficits.

Her clinical presentation was discordant with myasthenia gravis, and the acetylcholine receptor antibody panel was negative. A lumbar puncture was subsequently performed, revealing albumino-cytologic dissociation with a cerebrospinal fluid (CSF) protein count of 587 mg/dL and a white blood cell count of 4/cumm, confirming the diagnosis of Guillain-Barre syndrome (GBS) (Table 2). Zero oligoclonal bands were observed in the CSF, however, two paired bands were observed in both the CSF and serum, paired bands suggest an immune response to an inflammatory process outside the central nervous system (CNS) and are unlikely to represent a CNS demyelinating disease.

**Table 2:** Cerebrospinal fluid analysis shows elevated protein, but WBC count remains within normal limits, thus showing albumino-cytological dissociation. CSF- Cerebrospinal fluid; WBC- White blood cell; RBC- Red blood cell

Cerebrospinal Fluid Cell Analysis	Result	Ref Range & Units
Color, CSF	Colorless	
Clarity, CSF	Clear	
WBC, CSF	4	<=5 /cumm
RBC, CSF	15	<=5 /cumm
Neutrophil Count, CSF	12	<=10 %
Lymphocytes, CSF	68	<=70%
Mono+Macro, CSF	20	<=37 %
Glucose, CSF	71	40 - 70 mg/dL
Protein, CSF	587.2	15.0 - 45.0 mg/dL
CSF Culture	No growth at 72 hours	
CSF Gram Stain	No Bacteria seen	

The patient was started on Intravenous Immunoglobulin (IVIG therapy) on day third of hospital admission and day two of intensive care unit (ICU) admission for five days which showed a significant positive response as her neurological status started improving, and the patient was extubated eventually. The patient recovered significantly in the next few weeks and was successfully discharged to a short-term rehabilitation facility.

## Discussion

GBS is a prevalent cause of acute polyneuropathy in adults. International Guillain Barré Syndrome Outcome Study (IGOS) revealed that the majority of patients with Guillain-Barré syndrome (GBS) had a trigger event within four weeks of the emergence of neurological symptoms. Upper respiratory tract illness and gastroenteritis were identified as the two most identified triggers in the aforementioned research [5]. Consensus on the etiology of GBS indicates antecedent infections elicit an immune response with subsequent antibodies that cross-react with peripheral nerve epitopes, a mechanism known as molecular mimicry [6]. In our patient's instance, there was no history of diarrhea to indicate recent infection from *C. jejuni* which is the most common infectious trigger for GBS, furthermore, the fact that COVID-19 infection occurred within the past four weeks of the commencement of neurological symptoms indicates that SARS-COV-2 is the most probable trigger for GBS in her case. Our patient's acute onset of symmetric weakness in lower extremities that rapidly progressed to involve upper extremities, areflexia in lower limbs, severe respiratory muscle weakness requiring intubation and mechanical ventilation, negative lab workup for other common causes of acute polyneuropathies, time of symptom onset to nadir being less than four weeks, and CSF findings of albumino-cytologic dissociation satisfy the National Institute of Neurological Disorders and Stroke criteria for diagnosing GBS (Table 3). Within three days of beginning IVIG treatment, the patient's respiratory status improved dramatically and continued to improve at the post-acute short-term rehabilitation center.

**Table 3:** National Institute of Neurological Disorders and Stroke criteria for diagnosing GBS Recreated with reference from the National Institute of Neurological Disorders (NINDS) and Stroke criteria for diagnosing GBS [7].

NINDS diagnostic criteria for GBS
*Required features*:
Acute progressive weakness of the limbs, usually starting in the lower limbs and often progressing to involve but not limited to upper limbs, trunk, ocular muscles, bulbar and facial muscles.
Decreased deep tendon reflexes or areflexia
*Supportive features*:
Symmetric and bilateral symptoms
Autonomic dysfunction
Cranial nerve involvement
Pain in the lower limbs or trunk
Mild sensory dysfunction
Absence of fever
CSF showing albumino-cytologic dissociation
Electrodiagnostic abnormalities indicative of GBS
Quick Recovery in a few weeks after the halt of progression

## Conclusions

This case describes the course of GBS with preceding COVID-19 infection and its therapeutic response. While the severity of the GBS necessitated ICU care, the patient's response to IVIG treatment was remarkable. This case report reinforces the relationship between GBS and COVID-19 infection. It also necessitates being aware of neurological problems following COVID-19 infection and identifying them early to potentially halt the progression of the disease and improve patient care.

## References

[1] Rees JH, Soudain SE, Gregson NA, Hughes RA (1995). Campylobacter jejuni infection and Guillain-Barré syndrome. N Engl J Med.

[2] Hasan I, Saif-Ur-Rahman KM, Hayat S (2020). Guillain-Barré syndrome associated with SARS-CoV-2 infection: a systematic review and individual participant data meta-analysis. J Peripher Nerv Syst.

[3] Caress JB, Castoro RJ, Simmons Z, Scelsa SN, Lewis RA, Ahlawat A, Narayanaswami P (2020). COVID-19-associated Guillain-Barré syndrome: the early pandemic experience. Muscle Nerve.

[4] Fokke C, van den Berg B, Drenthen J, Walgaard C, van Doorn PA, Jacobs BC (2014). Diagnosis of Guillain-Barré syndrome and validation of Brighton criteria. Brain.

[5] Doets AY, Verboon C, van den Berg B (2018). Regional variation of Guillain-Barré syndrome. Brain.

[6] Yuki N, Hartung HP (2012). Guillain-Barré syndrome. N Engl J Med.

[7] (1978). Criteria for diagnosis of Guillain-Barré syndrome. Ann Neurol.

